# The effect of Self-Help Groups on access to maternal health services: evidence from rural India

**DOI:** 10.1186/1475-9276-12-36

**Published:** 2013-05-28

**Authors:** Somen Saha, Peter Leslie Annear, Swati Pathak

**Affiliations:** 1 Nossal Institute for Global Health, The University of Melbourne, Level 4, Alan Gilbert Building, 161 Barry St, Carlton, Victoria 3010, Australia; 2 Indian Institute of Public Health Gandhinagar, Drive in Road, Thaltej, Ahmedabad, Gujarat 380054, India; 3 Indian Institute of Management, Ahmedabad, Gujarat 380015, India

**Keywords:** Self help group, Institutional delivery, Family planning, Barriers, India

## Abstract

**Introduction:**

The main challenge for achieving universal health coverage in India is ensuring effective coverage of poor and vulnerable communities in the face of high levels of income and gender inequity in access to health care. Drawing on the social capital generated through women’s participation in community organizations like SHGs can influence health outcomes. To date, evidence about the impact of SHGs on health outcomes has been derived from pilot-level interventions, some using randomised controlled trials and other rigorous methods. While the evidence from these studies is convincing, our study is the first to analyse the impact of SHGs at national level.

**Methods:**

We analyzed the entire dataset from the third national District Level Household Survey from 601 districts in India to assess the impact of the presence of SHGs on maternal health service uptake. The primary predictor variable was presence of a SHG in the village. The outcome variables were: institutional delivery; feeding new-borns colostrum; knowledge about family planning methods; and ever used family planning. We controlled for respondent education, wealth, heard or seen health messages, availability of health facilities and the existence of a village health and sanitation committee.

**Results:**

Stepwise logistic regression shows respondents from villages with a SHG were 19 per cent (OR: 1.19, CI: 1.13-1.24) more likely to have delivered in an institution, 8 per cent (OR: 1.08, CI: 1.05-1.14) more likely to have fed newborns colostrum, have knowledge (OR: 1.48, CI 1.39 – 1.57) and utilized (OR: 1.19, CI 1.11 – 1.27) family planning products and services. These results are significant after controlling for individual and village-level heterogeneities and are consistent with existing literature that the social capital generated through women’s participation in SHGs influences health outcome.

**Conclusion:**

The study concludes that the presence of SHGs in a village is associated with higher knowledge of family planning and maternal health service uptake in rural India. To achieve the goal of improving public health nationally, there is a need to understand more fully the benefits of systematic collaboration between the public health community and these grassroots organizations.

## Introduction

As India strives to achieve universal health coverage, the main challenge is to expand coverage to all citizens with protection from the costs of basic health services. The poor generally have worse health outcomes and access to care compared to the non-poor. Poor health contributes to the persistence of India’s high poverty rates, with health expenditures driving 39 million families into poverty each year [1]. Even when treatment is sought, significantly smaller sums of money are spent on treatment of women than on men [2]. Gender discrimination exists in child feeding, health care, and nutrition status in India [3-9], and other South Asian countries [10,11].

Overcoming barriers to health service access is likely to be more difficult for the poor and other vulnerable groups as the costs of care, lack of information and cultural barriers impede them from benefiting from public spending [12,13]. Factors such as poverty, inadequate housing and lack of education are the social roots of morbidity in developing countries [14]. Health cannot be achieved without addressing these social determinants of health, and the answer does not lie only in the health sector [15-18]. Socioeconomic disparities are the major determinants of population health [19].

In this paper we analyse the effect of social capital, generated through women’s participation in community networks known as Self-help Groups (SHGs), on access to maternal health services, using data available from a large national survey in India. SHGs have emerged as a development strategy having a primary focus on poverty alleviation and empowerment of women. Structurally, a SHG is a small economically homogeneous affinity group of the rural poor coming together to form savings and credit organizations. Members deposit an amount regularly in a common fund to meet emergency needs and to provide collateral free loans decided by the group [20]. These small groups (10–20 members each) of predominantly rural women are well established in the country. Meeting the need for access to capital, specifically articulated by women during the United Nation’s Conference on Women and Development in Mexico City in 1975, SHGs can, in many ways, be considered the cornerstone of much of the well-established microfinance activity in India. Katz [21] defined self-help groups as: “Voluntary, small group structures for mutual aid and the accomplishment of a special purpose”. They are usually formed by peers who have come together for mutual assistance in satisfying a common need, overcoming a common handicap or life-disrupting problem, and bringing about desired social and/or personal change. They often provide material assistance, as well as emotional support; they are frequently cause-oriented and promulgate an ideology or values through which members may attain an enhanced sense of personal identity.

Major NGOs like the Self Employed Women’s Association, BRAC (a major development organization), and Grameen Bank in Bangladesh have engaged extensively in promoting health related activities through SHG participation. In India, organizations based on the Gandhian philosophy of self-reliance had already been popularized during the freedom movement in British India [22]. SHGs reflect a similar philosophy and provide an avenue for poor rural women to access the microcredit system. In the early 1990s, policymakers in India took notice of SHG growth and influence and established a countrywide SHG Bank Linkage Programme (SBLP). SBLP, promoted aggressively by the National Bank for Agriculture and Rural Development, links mature SHGs with the formal banking system. SHGs are linked to Regional Rural Banks (RRB), commercial banks and cooperative banks to access microcredit as a source of additional capital for the group members to supplement their savings. By establishing the *Swarnajayanti Gram Swarojgar Yojana* in 1999, the Government of India aimed to provide self-employment to millions of villagers. Poor families living below the poverty line were then organized into SHGs established with a mixture of government subsidy and credit from investment banks.

**Figure 1:**
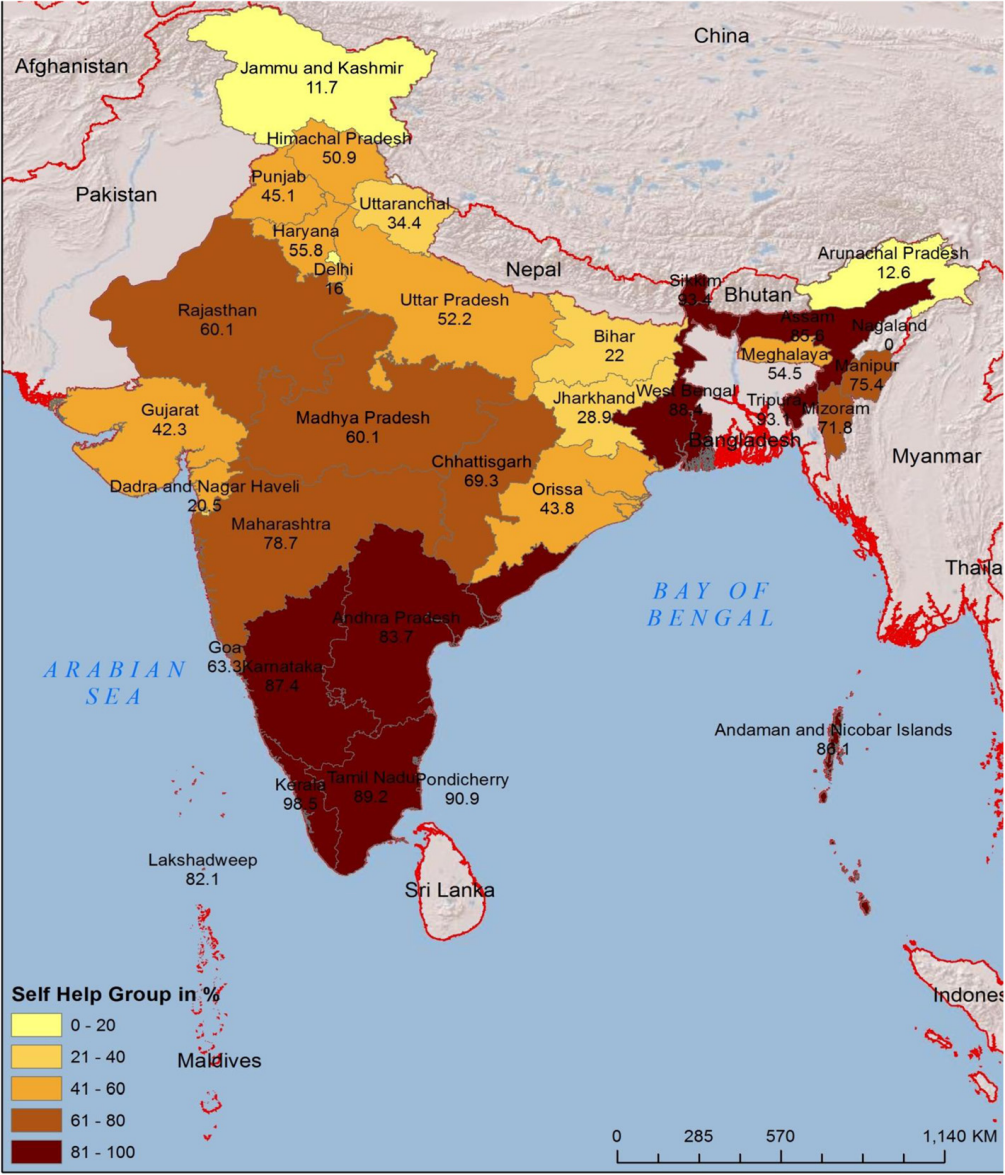
Villages in India with a SHG.

The main aim of these SHGs is to focus on income generation and raising poor families above the poverty line. The SHGs are supported and trained by non-government organizations (NGOs), community based organizations (CBOs), individuals, banks self-help promoting institutions, and microfinance institutions (MFI). The most prominent models of delivery for microfinance in India continue to be SHGs, promoted by the state governments, NGOs, a few regional rural banks, and specialized MFIs that use various models to make both group and individual loans [23]. The southern states of India experienced the largest concentration of SHG activities, both with state support, and promoted by private MFIs (Figure 1).

### Access to Health and SHG

The nature of SHG activities, where members meet regularly for transactions and training, creates solidarity and social capital. Social capital is built on features of social organization, such as trust, social norms and networks, that can improve the efficiency of society by facilitating coordinated actions [24]. The concept of social capital is further split into three connecting strands: bonding social capital (i.e. ties between immediate family members, neighbours and close friends); bridging social capital (i.e. ties between people from different ethnic, geographical, and occupational backgrounds); and linking social capital (i.e. ties between poor people and those in positions of influence in formal organizations such as banks and schools) [25]. SHGs, which bring village women together in a common organization for mutual support, are mobilized by existing bonding social capital, and then build linking social capital as the group members get involved in activities [26].

Several studies have found an association between social capital, generated from participation in microfinance, SHG, and participatory women’s groups on diverse health behaviours and health outcomes, as well as reducing inequity.

Globally there is emerging evidence to show that microfinance programmes have created non-financial benefits including improvements in health, hygiene and sanitation [19,27,28]. In post-tsunami Sri Lanka, a study using retrospective panel data from 350 randomly selected borrowers showed that microfinance loans provided after the disaster were instrumental in reducing the income gap between those who were hit and those who were not [29]. One analysis of a large dataset from three waves of the Indonesian Family Life Survey, showed a positive effect on changes in children’s health as MFI members were twice as likely to live in urban areas, have sewerage systems, regular garbage collection, electricity and better access to medical facilities [30]. Studies in India and Bangladesh have shown the positive effect of SHGs on reducing exclusion [31], improved childcare and contraceptive use [32,33].

In Maharashtra state, a project that trained women SHG members as health workers, initiated literacy programmes and provided funds for household health emergencies showed in the two decades after 1970 a reduction in infant mortality from 176 to 19 per 1000, a birth-rate decline from 40 to 20 per 1000, nearly universal access to antenatal care, safe delivery, and immunization, and a decline in rates of malnutrition from 40% to less than 5% [34,35].

A clustered randomized trial was conducted to assess the impact of a community mobilization programme through participatory women’s group among the indigenous communities of Jharkhand and Odisha states of India. The trial found newborn babies born to mothers associated with a women’s group significantly improved the likelihood of surviving within the first six weeks of their lives, compared to babies born to analogous households in control communities [36].

While available evidences, that includes rigorous randomised controlled trials and other rigorous methods, suggests the positive effect of social capital in reducing income gap, exclusion, saving newborn, and gender disparity in access to healthcare, Nayar [22] noted that most of the success stories from India are from large organization that incorporate self-help activities as part of other concurrent interventions. There has not previously been an assessment in India using nationally representative data.

The third round of the District Level Household Survey (DLHS-3), a national health survey conducted in 2007–08 in 601 districts of India included a question on the presence of a SHG in its village level questionnaire. Using this data, we are able to analyze the effect of SHGs on the knowledge and practices of women. This data provides the best available opportunity to analyze the influence of the presence of SHGs on women’s RCH knowledge and practices on a national scale. The results of our analysis are reported in this paper.

## Methods

Our study is the first to use national level data to analyse the impact of SHGs on health outcomes. We made a secondary analysis of data provided through the DLHS-3, which has been made publicly available. We analysed the national dataset, which was collected from 22,825 villages through the village questionnaire and from 643,944 ever-married women (15 – 49 years) through the ever-married women’s questionnaire. The DLHS-3 adopted a multi-stage stratified systematic sampling design that produced representative samples at national and state level after applying sampling weights to control for complex survey design [37]. The DLHS-3 was designed to provide information on family planning, maternal and child health, reproductive health of ever-married women and adolescent girls, and utilization of maternal and child healthcare services at the district level. At village level, the DLHS-3 included questions about the presence of SHGs in the village; unit level data from the village file and data from the ever-married women file were merged to conduct the analysis.

We analyzed the DLHS-3 dataset from 601 districts of India. Our hypothesis is that the presence of a SHG in a village is associated with improved access to maternal health services. Members of the groups are predominantly women, and maternal health indicators are good proxy indicators for overall health access. In this paper we have used four measures of women and child health knowledge and practices: institutional delivery; feeding new-born colostrum; knowledge about family planning services; and use of family planning methods. We measured knowledge and use of family planning by women who were aware of and practiced at least one of the following methods: female sterilization, IUD, oral contraceptive pills, emergency contraception and female condom. Indicators were transformed into binary measures by re-coding all ‘yes’ responses as 1 and ‘no’ as 0. For place of delivery: deliveries at hospital, dispensary, urban health centre/urban primary health centre, community health centre/rural hospital, primary health centre, sub-center, Ayush hospital/clinic, NGO/trust clinic, private hospital/clinic and on-way-to-hospitals were re-coded as 1, and delivery at home and work place were re-coded as 0. Data analysis was done using SPSS Version 19.

### Explantory and control variables

Pitt [38] identified three sources of bias in estimating cause-effect relationships: choice-based sampling, individual heterogeneity bias, and village heterogeneity bias. To address unmeasured individual and village attributes that affect both programme participation and health outcome, we instituted some controls. For individual heterogeneity we controlled for: respondent education (illiterate, primary, middle and higher secondary and above), wealth quintile, heard or seen health messages; and for village level heterogeneity we controlled: accessibility of Community Health Centre/Rural Hospital (CHC/RH), villages with any beneficiaries of JSY in last one year, and health and sanitation committee in village. Choice-based sampling is addressed by the sample size, and the national nature of the survey that can tease out the contribution of self-help groups independent of other concurrent activities or the organizational infrastructure. Table 1 shows the number and percentage distribution of responses by selected characteristics.

**Table 1:** Predictor and control variables used in the analysis

**Variable**	**Percentage**	**Number**
Predictor variable		
Village have SHG	57.9	13,211
Individual control variables		
Heard or seen health messages	85.9	553,225
Wealth Quintile		
Poorest	18.0	51,707
Second	21.9	62,996
Middle	25.0	71,732
Fourth	22.4	64,218
Richest	12.7	36,425
Mother’s level of education		
Illiterate	46.7	300,526
Primary (1-7)	23.5	151,048
Middle (8-10)	20.0	128,739
Higher secondary and above (11+)	9.9	63,631
Village control variables		
Health and sanitation committee in village	28.7	6,554
Accessible CHC/RH	77.4	16,609
Beneficiary of JSY	90.1	16,853

Numbers are unweighted.

### Statistical models

We computed forward stepwise logistic regressions adding different levels of control variables to a base model that regress our four outcome variables (institutional delivery, feeding colostrum, knowledge of family planning, and ever used family planning) on the availability of SHG in the village. We used a fixed effect unique to a district that captures the time-invariant differences across districts. For each of the four outcome variables, three models were estimated. In Model 1, the effect of presence or absence of a SHG in the village was modelled. This model represented the total variance in the four outcome variables with the presence or absence of a SHG. In Model 2 only individual level control factors (respondent education, work status, and heard or seen health messages) were included. In Model 3 individual and village background controls: village electrification, education facility available in the village, village connected through an all-weather road, distance from nearest hospital, beneficiaries of JSY in last one year, and health and sanitation committee were included. All models use survey weights to account for sample design and population weighting and standard errors are adjusted for clustering at the district level.

The focus of the analysis was the change in the coefficient of the presence of a SHG. The results are shown as odds ratios (ORs) with 95 per cent confidence intervals (CIs). The magnitude of the change was interpreted as the (exponentiated coefficient – 1.0) x 100. The small variance inflation factor of 1.09 (not reported) indicated the absence of any significant co-linearity between explanatory variables in the regression model.

## Results

### SHGs in India

As per DLHS-3 data, 57.9 per cent of Indian villages have a self-help group (Figure 1). The majority of these groups are located in southern and north-eastern India, followed by Maharashtra, Chhattisgarh, Rajasthan and Madhya Pradesh.

### Descriptive statistics

**Figure 2:**
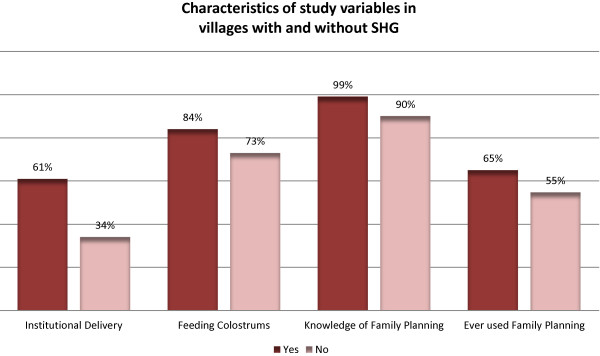
Study variables in villages with and without SHG.

The descriptive statistics (Figure 2) show some interesting findings on the four measures of women and child health knowledge and practices. The overall use of family planning was found to be very low. The presence of a SHG has a positive and strong correlation with all four measures of knowledge and practices. Compared to households in villages without a SHG, households in villages with a SHG are more likely to go for institutional delivery, more likely to feed new-borns colostrum, and more likely to have knowledge of and use family planning products and services. Members engaged in self-help activity feel a sense of connectivity and discuss issues ranging from place of delivery to feeding the baby and family planning products and services.

### Estimation results

As discussed in the methods sections earlier, we present our results on four measures of maternal and child health knowledge and practices: institutional delivery; feeding new-born colostrum; knowledge about family planning services; and ever used family planning.

*Predictor of institutional delivery* (Table 2): The presence of a SHG in a village is associated with 19 per cent higher odds of mother’s delivering in an institution (CI: 1.13 – 1.24), holding other variables constant. The reduction in odds from 1.30 to 1.19 in presence of individual and village level controls indicates the influence of other factors affecting the outcome. Model 2 adds individual control. The coefficients of individual control variables illustrate that mother’s education, wealth and having heard or seen health messages are important mediating pathways to influence institutional delivery. At the village level, the presence of a health and sanitation committee in the village, accessibility of CHC/RH (OR: 1.16), and beneficiary of JSY (OR: 1.30) are important mediating pathways that influence institutional delivery.

**Table 2:** Effect on institutional delivery

**Institutional delivery**	**Only predictor variable**	**Individual control**	**Full model**
Presence of SHG	1.30 (1.27-1.33)	1.26 (1.19-1.28)	1.19 (1.13-1.24)
Mother’s education		1.52 (1.50-1.54)	1.52 (1.50-1.54)
Wealth quintile		1.53 (1.51-1.55)	1.51 (1.49-1.53)
Heard or seen health messages		1.80 (1.73-1.88)	1.79 (1.72-1.87)
Health and sanitation committee in village			1.16 (1.12-1.19)
Accessible CHC/RH			1.16 (1.13-1.20)
Beneficiary of JSY			1.30 (1.25-1.36)
District fixed-effect	Yes	Yes	Yes
N	138,068	138,068	138,068

Figures are odds ratio with 95% Confidence Interval.

*Predictor of feeding colostrum* (Table 3): The presence of a SHG in a village is associated with 8 per cent higher odds of an increase in colostrum feeding. Mother’s education, wealth, and having heard or seen health message are important individual level mediating pathways, while being a beneficiary of JSY (OR: 1.32) is an important village level mediating factor predicting colostrum feeding. The presence of a health and sanitation committee in a village or accessibility of a CHC/RH does not appear to influence the outcome.

**Table 3:** Effect on colostrums feeding

**Colostrums feeding**	**Only predictor variable**	**Individual control**	**Full model**
Presence of SHG	1.20 (1.17-1.23)	1.09 (1.06-1.12)	1.08 (1.05-1.14)
Mother’s education		1.33 (1.31-1.36)	1.33 (1.31-1.36)
Wealth quintile		1.08 (1.07-1.10)	1.08 (1.07-1.10)
Heard or seen health messages		1.46 (1.41-1.51)	1.47 (1.42-1.53)
Health and sanitation committee in village			0.99 (0.96-1.02)
Accessible CHC/Rural Hospital			0.91 (0.88-0.95)
Beneficiary of JSY			1.32 (1.27-1.37)
District fixed-effect	Yes	Yes	Yes
N	135,823	135,823	135,823

Figures are odds ratio with 95% Confidence Interval.

*Knowledge about Family Planning (*Table 4): Households in villages with a SHG are at 48 per cent higher odds of knowing at least one modern family planning method. Model 1 produced an odds ratio of 2.13, indicating the strong influence of having heard or seen health messages in knowledge generation about family planning. More educated and wealthy women are more likely to have knowledge of family planning. Accessibility of a CHC/RH, and having been a beneficiary of JSY are village level variables influencing the outcome.

**Table 4:** Stepwise logistic regression of knowledge of family planning

**Knowledge of family planning**	**Only predictor variable**	**Individual control**	**Full model**
Presence of SHG	2.13 (2.01-2.26)	1.54 (1.45-1.63)	1.48 (1.39-1.57)
Women’s education		1.11 (1.06-1.16)	1.11 (1.06-1.16)
Wealth quintile		1.44 (1.40-1.49)	1.43 (1.39-1.47)
Heard or seen health messages		9.35 (8.80-9.94)	9.23 (8.68-9.81)
Health and sanitation committee in village			1.03 (0.96-1.11)
Accessible CHC/RH			1.35 (1.27 -1.43)
Beneficiary of JSY			1.29 (1.18-1.40)
District fixed-effects	Yes	Yes	Yes
N	397,055	397,055	397,055

Figures are odds ratio with 95% Confidence Interval.

*Ever-used family planning* (Table 5): Presence of a SHG is associated with 19 per cent higher odds of ever using family planning. Women’s literacy does not show a positive association with use of family planning, suggesting the lack of empowerment and decision making on reproductive choice. Wealth status, and heard or seen health message are important individual level factors, while accessibility of CHC/RH, and beneficiary of JSY are important village level mediating pathways influencing use of family planning.

**Table 5:** Stepwise logistic regression of using family planning

**Ever used family planning**	**Only predictor variable**	**Individual control**	**Full model**
Presence of SHG	1.20 (1.13-1.28)	1.21 (1.14-1.29)	1.19 (1.11-1.27)
Women’s education		0.77 (0.75-0.80)	0.77 (0.75-0.80)
Wealth quintile		1.24 (1.21-1.29)	1.24 (1.20-1.27)
Heard or seen health messages		1.36 (1.16-1.59)	1.35 (1.16-1.59)
Health and sanitation committee in village			1.06 (0.99-1.14)
Accessible CHC/RH			1.12 (1.03-1.20)
Beneficiary of JSY			1.20 (1.08-1.34)
District fixed-effects	Yes	Yes	Yes
N	19,143	19,143	19,143

Figures are odds ratio with 95% Confidence Interval.

### Limitations

As information on women’s actual participation in SHG activities was not included in the DLHS-3 dataset, our analysis provides an instructive but partial picture of the impact of SHGs on health outcomes. There are a number of limitations to our study. First, we did the analysis at the aggregate country level. This masks variations in the spread and intensity of SHG activity across India, as depicted in Figure 2 above. Secondly, the presence of a SHG in a village could only partially explain the level of activity. The level of women’s participation in a SHG [39], the availability of credit [40] and the duration of association [31,41] are other key predictors of health outcomes that the DLHS-3, due its limited scope and intent, did not address. Thirdly, we also did not find within the DLHS-3 manual an explicit definition of SHGs, or any distinction between the possible impact of SHGs and other women’s or community groups. The fourth limitation relates to the design and nature of the DLHS-3, including its reliance on self-reported information from respondents and the cross-sectional nature of the survey, as described by Jat et al. [42]. The survey collected the responses to the questionnaire only at their face value and had no opportunity to probe. Hence we could examine only the association between explanatory variables and four indicators of maternal health services uptake; we were not able to draw conclusions about causality. Nonetheless, ours is the first attempt to analyse these issues using a nationally representative dataset. Using this large national level dataset allowed us to address two important biases revealed in previous studies: choice based sampling [38] and teasing out the contribution of SHGs within the organizational infrastructure [22].

## Discussion and conclusions

Using a large national health survey data set from India, we examined the association between the presence of a SHG and maternal health service uptake measured through institutional delivery, feeding colostrum to new-born, knowledge and use of family planning (after controlling for individual and village level factors). Our study shows respondents from villages with a SHG were more likely to have delivered in an institution, fed new-born with colostrum, and known about and utilized family planning products and services. These groups give the communities an avenue to voice their concerns and provide a unique space in which solidarity is created through promoting shared visions and goals and combining collective strengths. The presence of trust and social capital empowers communities and positively influences individual and community health. However, on their own, SHGs can have only limited impact. This is explained by the relatively low odds ratio in presence of individual and village level controls. Clearly, in order to have maximum impact on community health, there is a need for additional complementary health programmes to build on the solidarity and social capital generated as a result of the group.

The study adds to the evidence that trust, solidarity and sense of belongingness as a result of participation in a SHG are important determinants of health outcomes. Additionally, by using a large national health survey dataset, our study shows this effect is independent of organizational infrastructure. There is a strong case for policy makers to work closely with these groups and leverage on their strengths for health improvement and poverty reduction.

By linking the activities of SHGs to broader programmes, such as the National Rural Health Mission (NRHM), Indian policy makers could increase the impact of these proven interventions designed to provide improved access to health care and address poverty. Programs like the NRHM could effectively use the SHG community structures to promote awareness and generate increased demand for services. The NRHM would benefit by linking with the range of services provided in the community by both the individual SHGs and their federated structure. These services include creating community awareness, promoting institutional delivery, childhood immunization, preventive care and lay counselling through village health and sanitation committees, community monitoring, emergency health loans and health savings funds, and the provision of low-cost health products, such as sanitary napkins, contraceptive choices and first-aid care at the community level. Even so, scaling-up such programmes to national level must be based on reliable evidence related to implementation procedures to avoid difficulties that were previously experienced with the Jamkhed, Kakamega and other similar experiments [34].

This has implications for low- and middle-income countries where barriers to access to health services, including informational and cultural barriers, prevent poor and vulnerable groups from benefiting from public health spending. With a global outreach to 205 million microfinance members [43], these groups are an innovative way to combine poverty alleviation and community health interventions into an integrated strategy that leverages existing resources to achieve greater impact and scale. Finally we conclude that to achieve the goal of improving public health, there is a need to better understand the benefits of systematic collaboration between the public health community and these grassroots organizations.

## Competing interests

The authors declare that they have no competing interests.

## Authors’ contributions

SS led the drafting of the manuscript, and contributed to all aspects of the study. PA participated in conceptualization of the study design, and advised on most aspect of the study. SP provided support for statistical analysis, and drafted part of the manuscript. All authors have read and approved the final manuscript and declare no competing interest.
